# Low serum iron as a risk factor for ICU-acquired bacteremia: study of a large cohort database

**DOI:** 10.1186/cc14150

**Published:** 2015-03-16

**Authors:** S Fernandes, D Bruno, J Morgado, C Calle, C Ferreira

**Affiliations:** 1 Centro Hospitalar Lisboa Norte CHLN, Lisbon, Portugal; 2 Hospital Fernando Fonseca, Lisbon, Portugal; 3 Centro Hospitalar de Lisboa Central, Lisbon, Portugal; 4 Instituto Português de Oncologia, Lisbon, Portugal; 5 National Institute of Legal Medicine and Forensic Sciences, Coimbra, Portugal

## Introduction

Bloodstream infections in the ICU are a major trigger of morbidity and mortality. Several risk factors for bacteremia have been previously identified, such as presence of a central venous catheter or invasive ventilation [1,2]. Iron is a key element for bacteria growth, and its metabolism is extensively altered by inflammation. We aim to determine whether iron deficiency is a risk or protective factor for bacteremia in the ICU.

## Methods

We performed a retrospective analysis of patients included in the MIMIC-II database, an ICU database that collected data from patients admitted to the medical, surgical, coronary and cardiac surgery ICU of Boston's Beth Israel Deaconess Medical Center during a period of 7 years. We performed logistic regression models to assess the association between iron and bloodstream infection.

## Results

We included 3,980 patients, 2,988 with low serum iron (<60 ng/ ml) and 992 with normal/high serum iron (≥60 ng/ml). During their first stay in the ICU, 351 (8.82%) patients developed bloodstream infections. Low serum iron was associated with increased odds of bloodstream infection (OR: 1.37; 95% CI: 1.04 to 1.80). After adjusting for age, gender, Simplified Acute Physiology Score, presence of central venous catheter, ICU type, transfusions performed before iron measured, neoplastic disease, diabetes mellitus, hepatic disease, congestive heart failure and ferritin levels, low levels of iron were still associated with an increased odds of bacteremia (OR: 1.41; 95% CI: 1.03 to 1.9). In contrast, low serum iron was associated with a decreased risk of death in the hospital (adj OR: 0.73, CI: 0.57 to 0.95).

## Conclusion

Low serum iron increases the risk of bloodstream infection in the ICU, and should be considered as a risk factor to stratify patients' risk of bacteremia during ICU stay.
